# The PROMoting the USE of SWATs (PROMETHEUS) programme: Lessons learnt and future developments for SWATs

**DOI:** 10.1177/26320843221089632

**Published:** 2022-06-10

**Authors:** Laura Clark, Catherine Arundel, Elizabeth Coleman, Laura Doherty, Adwoa Parker, Catherine Hewitt, David Beard, Peter Bower, Paul Brocklehurst, Cindy Cooper, Lucy Culliford, Declan Devane, Richard Emsley, Sandra Eldridge, Sandra Galvin, Katie Gillies, Alan Montgomery, Chris Sutton, Shaun Treweek, David Torgerson

**Affiliations:** 1 York Trials Unit, Department of Health Sciences, 8748 University of York, York, UK; 2 Nuffield Department of Orthopaedics, Rheumatology, and Musculoskeletal Science, NIHR Biomedical Research Unit, 6396 University of Oxford, Oxford, UK; 3 National Institute for Health Research School for Primary Care Research, Centre for Primary Care and Health Services Research, University of Manchester, Manchester, UK; 4 1506 Bangor University, Gwynedd, UK; 5 School of Health and Related Research, 7315 University of Sheffield, Sheffield, UK; 6 Bristol Trials Centre, Clinical Trials and Evaluation Unit, 1980 University of Bristol, Bristol, UK; 7 School of Nursing and Midwifery, 8799 National University of Galway, Galway, Ireland; 8 Department of Biostatistics and Health Informatics, 4616 King’s College London, Institute of Psychiatry, Psychology and Neuroscience, London, UK; 9 Institute of Population Health Sciences, 4617 Queen Mary University of London, London, UK; 10 Health Services Research Unit, 1019 University of Aberdeen, Health Sciences Building, Foresthill Aberdeen, Aberdeen, UK; 11 Nottingham Clinical Trials Unit, 6123 University of Nottingham, Nottinghamshire, UK; 12 School of Health Sciences, 5292 University of Manchester, Manchester, UK

**Keywords:** study within a trial, methodological, recruitment and retention, research designs and methods, randomised controlled trial

## Abstract

**Introduction:**

The PROMETHEUS programme (PROMoting THE USE of SWATs) was funded by the UK Medical Research Council (MRC) and Clinical Trials Unit (CTU) infrastructure funding from the National Institute for Health Research (NIHR). The purpose was to develop strategies to increase the recruitment and retention evidence base. This paper aims to present observations from this work.

**Observations:**

The PROMETHUS programme funded 42 SWATs, the average cost of each SWAT was £4007. A central coordination point enabled a concentrated effort in SWAT research activity leading to a rapid increase in the evidence base. The methodological feasibility of undertaking a coordinated SWAT design was established. The international Trial Forge SWAT Network was developed in 2021 to connect research groups in response for the need to connect teams undertaking methodological research. A SWAT reporting template and a database of researchers willing to peer review SWATs are also needed to improve the reporting of SWATs.

**Discussion:**

There is a need to develop a strategy to aid teams to identify a suitable SWAT for their host trial populations and a mechanism to communicate SWAT research priorities. Work is needed to increase the awareness of the methodological importance of SWAT research with research teams and develop engagement strategies to increase SWAT activity. Continued collaboration with the HRA is necessary to refine the SWAT approvals process.

**Conclusion:**

The coordination PROMETHEUS provided is crucial to increasing the recruitment retention evidence base. The Trial Forge Network will be key to provide ongoing networking and dissemination opportunities.

## Introduction

Randomised controlled trials (RCTs) are crucial for providing evidence-based healthcare. However, trials frequently fail to recruit on time and budget, and they often experience significant attrition of recruited participants.^1^ Strategies used by trialists to improve recruitment and retention (the top methodological research priorities identified by UK Clinical Trials Units’ directors^2^) have often not been rigorously evaluated. Studies Within a Trial (SWATs) are one method to assess the effectiveness of recruitment and retention strategies.^3^ The MRC funded PROMETHEUS programme – PROMoting THE USE of SWATs (Grant number MR/R013748/1) – aimed to rapidly increase the evidence base around recruitment and retention strategies.

The PROMETHEUS programme was conducted between 2018 and 2021 and offered UK trial teams up to £5000 to embed a SWAT within their host trial, alongside methodological support. Twelve Clinical Trials Units (CTUs) applied for PROMETHEUS funding, 42 SWATs were funded and embedded in 31 different host trials across 17 different areas of health research, which is to date is the biggest single effort to generate SWAT evidence worldwide. This activity represents a substantial increase in the global methodological evidence base: PROMETHEUS will add 12 more SWATs to the Cochrane systematic review of recruitment interventions, an increase of 18% (12/68)^4^ and 30 SWATs to the Cochrane review of retention interventions, an increase of 79% (30/38).^5^ Most SWATs focussed on the potential or enrolled participants, such assessing changes to participant information leaflets, whilst others focussed on training staff recruiting participants. Each team was asked to publish the results of their SWAT. PROMETHEUS also funded and completed two simultaneous SWATs evaluations.^6,7^ Many of the PROMETHEUS funded SWATs are still ongoing, due to the COVID-19 pandemic pausing host trials. Further details of the PROMETHEUS programme are available here.^8^ Following the success of PROMETHEUS, additional funding was received from the National Institute Health (NIHR) (award ID: NIHR132547). This funding extended the project and enabled the PROMETHEUS group to continue work to investigate how teams, within the UK, can be better supported to implement recruitment and retention SWATs. The PROMETHEUS programme was the first of its kind which enabled a range of observations on the coordination and conduct of SWATs to be identified. The lessons learnt and experience gained throughout the PROMETHEUS programme about SWAT research are largely based within the context of the UK; however, these should be transferable to other settings. The aim of this discussion paper is to:

- Discuss the observations and conclusions from the original programme (Grant number MR/R013748/1) and that as part of the additional funding received (award ID: NIHR132547);
- Consider the focus of future research and strategies identified from this body of work that could lead to an increase in the recruitment and retention evidence base.

## Original programme observations (Grant number MR/R013748/1)

### Funding

PROMETHEUS was able to offer teams funding which is typically a limiting factor to researchers undertaking a SWAT.^9^ The average cost of a SWAT funded through PROMETHEUS was £4,007, lower than the anticipated £5,000, which enabled more SWATs to be funded. See the PROMETHEUS results paper for further details of these costs.^8^

There are limited funding streams available for methodological work. However, since the PROMETHEUS programme was conceived, the NIHR Health Technology Assessment (HTA) programme have also begun to offer UK research teams up to £10,000 to embed a SWAT^10^ and up to £30,000 to embed studies within a project across a number of their funding streams for multiple long-term conditions.^11^ Our experience with PROMETHEUS suggests that this level of funding should be suitable for many different SWAT interventions to be embedded within host trials. However, within the PROMETHEUS programme lower cost and easier SWATs were chosen by the PROMETHEUS Trial Management Group as priorities. Unfortunately, current figures from the NIHR are not available and so it is not possible to identify the uptake of the NIHR funding. Teams have, however, noted that they often have not had the time to put resources into designing a SWAT. Additionally, teams have stated they do not want to complicate further an already complex study application and so have not accessed this funding. Funders could promote the priority SWAT questions within their grant application processes, which may remind and encourage trialists to include priority SWATs in grant applications. This may lead to an increase in applications to the available funding.

Once the MRC funded element of the PROMETHEUS programme ended, some trial teams stated they would be unable to embed further SWAT without external funding. As noted previously this has been partly addressed by at least one funder (NIHR) who allows up to £10,000 for a SWAT when included in a ‘standard’ research application. For RCTs funded elsewhere then choosing a low-cost intervention to evaluate could be a solution. For example, many trials routinely send newsletters to patients in the belief this improves retention. SWATs of different types of newsletters could be relatively easily, and cheaply, undertaken. There are similar other low-cost SWATs (e.g. electronic reminders via email or text). Arundel et al. offer a further discussion around funding SWATs.^12^

There needs to be a full cost breakdown available for SWAT interventions that would aid teams to apply for funding. Few SWATs are cost free nor free from a time burden; a method to communicate all aspects of undertaking SWATs in a transparent way to teams is recommended. Trial Forge evidence packs^13^ are being designed to do this. Our findings support their continued use and implementation. The PROMETHEUS team have contributed to evidence packs on the use of electronic prompts and pens for retention.

### Coordinating centre

PROMETHEUS has been key in providing support to teams and has been a central coordination point for SWAT design and delivery. The most support given was at the set-up stage, particularly around identifying a SWAT intervention that would be both suitable and acceptable within a specific host trial. Support was also given to teams to aid develop protocols and statistical analysis plans. Advice on ethical application was also given and, in some instances, undertaking statistical analyses for SWATs and writing up for publication.

Having a central contact also enables meta-analyses to be coordinated when sufficient SWAT replications have been undertaken leading to an increase in the evidence base. This is because they can monitor and will be aware of SWAT activity and can collate and synthesise the evidence when possible. The output from the PROMETHEUS programme will contribute to several meta-analyses, including the timing of electronic reminders on questionnaire return.^14^ Maintaining this point of contact is also important to promote and support further SWAT activities for example further replications if required by the findings of the meta-analysis. This has enabled a concentrated effort in SWAT research activity which has led to an increase in the evidence base.

The PROMETHEUS group collaborated with other trial methodology working groups such as Trial Forge,^15^ the Irish Health Research Board Trials Methodology Research Network (HRB-TMRN),^16^ and the Trial Conduct Working Group of the MRC-NIHR Trials Methodology Research Partnership.^17^ These pro-active collaborations with other groups promoting the use of SWATs enables more efficient systems to identify strategies to support teams embedding SWATs. Strategies have been identified through direct feedback when meeting with teams undertaking or enquiring about a SWAT and through feedback following the PROMETHEUS webinars. Discussions amongst the collaborators and different research partnerships that are directly involved in methodological research have also identified common barriers and facilitators to completing a SWAT. In turn, this is leading to discussions regarding the best actions needed to support research teams. Collaboration with these groups enables experiences and opportunities to be shared that could lead to identifying funding options and other mechanisms to provide support to teams. Areas that we have identified that are crucial to continuing the encouragement of SWAT work include the following: mechanisms to identify what a suitable SWAT intervention would be for a given host trial, a comprehensive repository of information for researchers undertaking SWATs, training webinars disseminating and discussing SWAT research and setting up a specific group to support SWAT activity. The CTU infrastructure funding obtained (award ID: NIHR132547) enabled further exploration of this, discussed later in this manuscript.

### Coordinated SWATs

As part of the PROMETHEUS programme two coordinated SWATs were undertaken.^8^ This is where one SWAT intervention is tested within multiple host trials in a centrally coordinated way within a limited time window. One assessed the effectiveness of sending a Christmas card on participant retention across eight host trials, run by two trials units (SWAT 82 in the SWAT repository^18^),^6^ and the other one recruiter training on recruitment rates across four host, surgical, trials.^7^

This design is efficient as it results in a rapid increase in the evidence base and has the possibility to answer SWAT questions definitively in one concurrent evaluation. Additionally, as one team coordinates the research activity the SWAT is easier to operationalise. These two coordinated SWATs have shown that these simultaneous designs are feasible and well received amongst research teams. This finding has led to the development of further coordinated SWAT evaluations assessing personalised SMS (compared to standard SMS) on retention (SWAT 35 in the SWAT repository^18^) and the addition of a pen with participant recruitment packs on recruitment rates (SWAT 37 in the SWAT repository^18^). Such SWATs benefit from the existence for a coordinating centre for the reasons as outlined above.

### Low engagement

Although 42 SWATs were funded there may be some lessons from understanding why some CTUs had lower levels of engagement than others. To be fully successful the ethos of the SWAT has to permeate to all units and institutions involved in formal evaluation of healthcare. There are several potential, and mainly anecdotal reasons why some units engaged less than others despite conducting similar work. These include workload and prioritisation – some units with a busy trial portfolio and caseload found it difficult to create time and space to address the objectives of PROMETHEUS, despite best intentions. Review of the topic matter of the SWAT also may have disincentivised some units. Although having high potential importance to getting a successful trial completed, the SWAT research question may have seemed less exciting and engaging than the trial research question itself. This could also be expressed by the Chief Investigator (CI), driving interest away from a potential SWAT.

There may be no incentive for a team to embed a SWAT within their host trial. For instance, although each SWAT should produce a peer reviewed paper in terms of contributing to a Research Excellence Framework it is unlikely any individual SWAT publication will form part of an institution’s submission. Uplift of the importance of SWATs is necessary to avoid this in the future, with senior institutional management needing to fully engage with SWAT programmes. The cross section of staff characteristics may also affect uptake of SWAT studies, or even in the case of a larger methodological SWAT assessment such as PROMETHEUS. Units with staffing profiles which perhaps have greater specialised expertise (such as Health economists or Statisticians) rather than trial delivery personnel, may have less inclination or capacity to explore ‘coalface’ methodological interventions such a recruitment or retention SWATs.

### Dissemination

There was great interest in the PROMETHEUS programme from trial teams applying for funding, which created collaboration and networking opportunities. A dissemination conference was planned for June 2020 which was moved to a webinar due to the COVID-19 pandemic. The webinar topics comprised the following: an overview of the PROMETHEUS programme, the collaboration between PROMETHEUS and Trial Forge,^19^ where additional evidence of recruitment and retention strategies was needed, the practicalities of implementing a SWAT and the support that the PROMETHEUS group could offer to research teams. The webinar was joined by approximately 150 attendees from seven countries including representatives from funding bodies. Due to its success, it was deemed that online presentations are an effective way to disseminate methodological research and engage with researchers.

## Clinical Trials Unit (CTU) infrastructure funding: Observations (award ID: NIHR132547)

Following completion of the MRC Award, additional funding was awarded to the PROMETHEUS group by the NIHR through its CTU Infrastructure funding to increase SWAT activity, publications and dissemination. A range of strategies were developed based on the experiences from the original PROMETHEUS programme through discussions with different trial teams. This funding differed from the MRC funding as the PROMETHEUS group could no longer provide funding to teams to embed SWATs.

### Webinar series

Following the success and reach of the dissemination webinar held as part of the original programme of work, we developed of a series of ‘PROMETHEUS hosted webinars’. Throughout the duration of the PROMETHEUS programme as well as collating common barriers and facilitators to SWATs reported here, we collected information to plan resources to support teams embedding SWATs. The purpose of this was to identify training or tools needed, and to elicit themes and topic areas for presentation and discussion at future webinars. The webinars also provided an opportunity for networking and feedback. We further developed the webinars through surveying attendees and direct discussions with trial teams, which subsequently identified a need for webinars in the following areas, see Table 1 for details, and the PROMETHEUS group webpage for webinar recordings.^20^

**Table 1.** Details of the PROMETHEUS webinar series.

Webinar title and date held	Details
*‘The Implementation and management of studies within a trial’* *September 2020*	Attendees requested the opportunity to hear examples of SWATs undertaken by different research teams with a focus on practical implementation. This webinar comprised of nine speakers presenting their SWAT research with an emphasis on the practicalities of how they implemented their SWAT.
‘*Recruitment and Retention Research priorities webinar’* *April 2021*	We collaborated with the Trial Methodology Research Partnership (TMRP) and Trial Forge and presented the current SWAT research priorities. The TMRP presented their mapping work to identify gaps in the recruitment and retention evidence base. We presented two SWATs that had been identified as a priority: Pens for recruitment and personalised SMS for retention. We provided protocols for teams to use to embed these SWATs within their own host trials
*‘Researcher experience webinar’* *October 2021*	Two researchers presented their methodological work. One, on their experiences on conducting SWATs in low middle-income countries and the other on their work around the use of digital tools in the recruitment and retention of participants
*‘Effective recruitment and retention’* *December 2021*	Two researchers presented their work exploring the importance of effective recruitment and retention methods, and presented methodological innovation in the form of the simultaneous/coordinated SWAT design, which can lead to a rapid increase in the evidence base

### Networking and the Trial Forge SWAT Network

SWAT research is fundamentally collaborative because of the need for replication evaluations. The findings of which are then included in a meta-analysis to determine the effectiveness of an intervention. For this to occur efficiently, there needs to be networking and communication between different research teams who are performing SWATs. Coordination and networking ensures that SWATs on the same topic are more homogenous, by ensuring that the intervention is similar, and identifying different patient populations where replication is needed to maximise generalisability.

We identified that there was a need for a formal network to link together teams globally that are undertaking SWAT research. In collaboration with Trial Forge, the PROMETHEUS group recently set up the ‘Trial Forge SWAT Network’.^21^ This Network enables research teams already working on SWATs to register their institution as a Network member. This Network initiative aims to improve communication, address challenges, support uptake of results, and present collaborative opportunities to teams worldwide to identify research gaps and improve the trial process evidence base. Currently the network has over 20-member research groups from the UK, Ireland, Australia and Iran. Individual meetings have been held with teams to discuss their own experience with methodological research and SWATs, including a discussion on the barriers they have encountered and suggestions to facilitate future SWAT research. The network comprises of Trial Managers and coordinators, statisticians, trial methodologists and Chief Investigators, which is the largest body of collaborators assembled with a focus on SWATs to date. We are using knowledge gained from these meetings to help plan future webinars and discuss agenda for further SWAT research. More information on joining the Network is available here.^21^

The Northern Ireland SWAT repository^18^ also shares SWAT protocols, which offers an opportunity for trialists to obtain the details of specific SWATs. Where SWATs have been evaluated and included in Trial Forge Evidence Packs, these also detail key components of design and delivery to enable broader implementation of strategies found to be effective.^13^ Furthermore, the wider research community can assist with supporting colleagues to undertake SWATs. When a team has undertaken a SWAT, sharing the materials they used in a central repository would benefit the wider research community and facilitate more rapid SWAT research. We hope that the Trial Forge SWAT keywork will play a central role in this coordination.

### Reporting and reviewing SWAT publications

Through discussions with researchers, many described difficulties with reporting and publishing SWATs, including the challenges in identifying suitable SWAT reviewers. Firstly, reviewer feedback had sometimes focussed on SWATs being underpowered and not including a sample size calculation, suggesting lack of knowledge or mis-understanding around the methodological features of SWATs. Secondly, the criteria for reviewers that journals specify can lead to those who have SWAT knowledge being ‘under qualified’. For example, some journals currently have a high barrier to peer review, such as requiring peer reviewers to hold a PhD and to have a minimal number of relevant publications, which more junior researchers may not have. As recruitment and retention SWATs are a niche methodological area, this results in fewer researchers being eligible to be reviewers. Reviewers may have also collaborated with authors previously, which decreases the number of reviewers further. To increase the pool of reviewers and speed up the reviewing process, we have developed a database of reviewers for SWAT publications.

Funding was a specific challenge to publishing SWATs, as was having dedicated time to write the publication and being able to prioritise this. To try to minimise the impacts of funding, we worked with the reporting platform F1000 to discuss their publication policies and have developed a ‘SWAT collection’.^22^ This platform has the benefit of being of lower cost than many other open access publishers and provides immediate public access while peer review is ongoing. We are currently collaborating with Trial Forge and have developed and are now piloting a reporting guideline, this will be made available for use in due course.

### Approval processes

Some research teams expressed frustration with the approval processes in the UK. It has been reported some had difficulty in gaining ethical approval for a replication SWAT, which, has already been undertaken and received previous ethical approval. A more straightforward and rapid approvals process is necessary for SWATs. The UK’s Health Research Authority (HRA) have identified this barrier. We are currently developing a new process with the HRA in collaboration with Trial Forge to streamline the ethical approval processes for SWATs.

### Identifying and selection of a suitable SWAT intervention

We identified that support is needed for research teams to aid identifying and selecting SWAT interventions. This support is particularly needed to aid identifying a SWAT which is a best fit with the host trial population, design or processes. We suggest that the optimal way to operationalise this support is to identify effective ways to communicate which SWAT interventions, including recruitment, retention, and other methodological interventions, that are a best fit for specific trial characteristics. The Cochrane reviews offer a clear starting point for this but as these are only updated every few years. There is a need to establish a more routine and consistent real-time method would enable the research gaps to be identified to enable more coordinated and rapid increase of the evidence base.

A further strategy could be to ensure there is a clear list of SWAT research priorities that includes both the SWAT intervention details and trial area for both funders and research teams to reference. This list would need to be updated as SWATs are performed to reflect the gaps in the evidence base. Boxall et al. have undertaken work on mapping existing SWATs in the SWAT repository^23^ to the PRIORITY questions.^24^ In addition, clarity on where additional replications of a SWAT is needed, and in which populations may be useful to guide coordinated delivery and so obtain conclusive answers to these questions.

There is however a need for pragmatism, particularly when evaluating whether additional SWAT replications are necessary. Additional replications use resources, take time and could result in a delay in a recommendation of a given strategy. The balance is ensuring that there is or is not confidence around the effectiveness of, or lack of, a strategy against advising on whether additional replications are really necessary. An example is using Short message service (SMS), where this electronic prompt is used to encourage questionnaire return, further replications of this SWAT have been recommended.^25^ Many teams already use SMS, there have been five replications of this retention strategy with the results suggesting they are an effective method to aid questionnaire return, with an increase of questionnaire return of 6.3% (95% CI of 0.5–12.2%).^26^ This therefore raises the question of whether it is a worthwhile use of SWAT resources to be suggesting further replication SWATs should be performed. Instead, a pragmatic judgement could be implemented meaning that this strategy is recommended so that trial teams can use this strategy confidently. However, SWATs on electronic reminders are still required to identify the optimum message content of an electronic reminder. A further option would be to undertake a value of information economic analysis. Here, the costs of undertaking additional replications of a SWAT are considered and compared to whether the costs of these additional replication would exceed the value of the information they would produce. Both of these options enable resources and research to be directed to assessing the effectiveness of a strategy where the evidence is not clear.

## Discussion

The PROMETHEUS team has begun to increase the conduct of SWATs and has identified a range of areas where further development is warranted. The conduct of 42 SWATs during this time enabled identification of key elements which require further development and coordination. This is essential to ensure that SWATs continue to be undertaken with sufficient repetitions available to enable conclusive meta-analyses to be performed. This will lead to an increase in the evidence base enabling identification of effective or ineffective recruitment and retention strategies enabling trialists to design and undertake efficient research.

We list here the findings and future priorities that we have identified through our work:

- The PROMETHEUS Programme has demonstrated that coordination of activity remains crucial to the delivery of SWATs and a central coordination point needs to continue.
- A strategy is needed to aid teams to identify and select suitable SWAT interventions for their host trials.
- SWAT priorities need to be clearly communicated to trial teams and funders.
- Pragmatic decisions should be taken when deciding if further SWAT evaluations are necessary.
- Economic evaluations are an area that should be explored to help inform future SWAT priorities, including undertaking value of information analyses.
- Working with trial teams to develop engagement strategies to undertake SWATs would be beneficial.
- The approvals process for SWATs needs streamlining, continued collaboration with the HRA is necessary to refine the process.
- Webinars are an effective tool to communicate methodological research which could continue to be utilised in the future.
- The Trial Forge SWAT Network has been well received by teams and will offer opportunities to share, disseminate and increase methodological research.

## Conclusion

Following the success of the PROMETHEUS programme, the priority is to continue to promote SWATs to enable the gaps in the knowledge base to close and further our understand of recruitment and retention issues in trials. The central coordination aspect that PROMETHEUS offered has been very effective in providing support to teams as well as organising webinars and networking opportunities. The PROMETHEUS Programme remains active and so there is an opportunity for trialists to continue to benefit from the repository of information developed during this programme. The Trial Forge SWAT Network will be key in continuing networking and dissemination of SWAT research.
